# Editorial: Effects of exercise intervention on emotional health and emotional processing: evidence related to psychology and physiology

**DOI:** 10.3389/fpsyg.2024.1474722

**Published:** 2024-08-30

**Authors:** Hongying Fan, Shiyi Ma

**Affiliations:** ^1^School of Psychology, Beijing Sport University, Beijing, China; ^2^Key Laboratory of Exercise and Physical Fitness (Beijing Sport University), Ministry of Education, Beijing, China; ^3^Laboratory of Sports Stress and Adaptation of General Administration of Sport, Beijing, China

**Keywords:** physical activity, emotional health, body mind relationships, exercise intervention, movement

The benefits of exercise on emotional health and emotional processing are gaining significant attention in both psychology and physiology. It is widely accepted that exercise constitutes an effective way in managing one's health (Mahindru et al., 2023). Recent studies demonstrate various promotion effects of physical activity, such as reducing anxiety and depression (Kandola and Stubbs, 2020). These benefits could prove advantageous in alleviating mental stress and enhancing overall life satisfaction. Although there are many benefits of exercise on mental and physical aspect, how exercise interventions work at both the physiological and psychological levels was a Research Topic that needs to be explored in depth.

For the general population, this Research Topic revealed the combined effects of other factors with exercise intervention. First, the study on university freshmen highlights the pivotal role of subjective exercise experience in predicting self-efficacy, interpersonal relationships, and anxiety among university freshmen. It underscores the chain-mediated effects where self-efficacy and interpersonal relationships modulate the impact of exercise on anxiety. These insights provide a theoretical foundation for developing targeted interventions to improve the mental health of university students (Xiang et al.). Second, the research exploring the moderated mediation model elucidates how physical activity enhances executive functions through the mediating roles of self-efficacy and negative emotions. The findings advocate for regular exercise among students to boost self-efficacy, reduce negative emotions, and consequently improve cognitive performance (Zhao et al.). Besides university students, research has also been conducted on normal adults. Physical activity was found to positively correlate with body and sexual self-esteem, which in turn enhanced marital satisfaction. Conversely, psychological distress negatively affected marital satisfaction. These findings highlight the complex interplay between physical self-perception, emotional well-being, and relationship quality, suggesting that regular physical activity can be a valuable component of marital satisfaction interventions (Snani et al.). Furthermore, the gender differences in exercise intervention can help formulate professional and scientific workout plans for different genders. This study examined how gender influences the relationship between personality traits, emotional intelligence, and physical exercise. It found that while males benefit from extended exercise duration through enhanced general mood and extraversion, females' exercise duration is negatively impacted by depressive symptoms. The results emphasize the need for gender-specific strategies to encourage sustained physical activity (Campos-Uscanga et al.). The last study extends the benefit of exercise intervention to auditory stimuli, finding that the sound of tennis helps alleviate anxiety. This innovative study investigated how the sound of tennis batting influences anxiety levels, with participants exposed to the sound experiencing a significant reduction in anxiety, highlighting the potential of sound-based therapeutic strategies (Wang et al.). Research consistently shows that exercise combined with other factors significantly benefits mental health and cognitive functions. Future studies could explore merging factors in this Research Topic. Specific exercise programs positively influence certain mental disorders, for example, yoga seems particularly effective in altering perception of bodily signals, cardiac activity, and emotion processing immediately after exercise (Herbert, 2022).

This Research Topic has filled the gap in understanding how exercise intervention affects emotional health and processing both psychologically and physiologically. On the neurological dimension, a review of EEG neurofeedback studies suggests that EEG neurofeedback can teach athletes to control their mental states, leading to better cognitive, emotional, and physical outcomes. Despite its promise, the review identified significant gaps, including inconsistent EEG target selection and a lack of robust control analyses. Future research should focus on standardizing protocols and validating the efficacy of neurofeedback training in sports (Cheng et al.). For the athletic group, a controlled study with competitive swimmers demonstrated that tapering strategies—characterized by a gradual reduction in training volume—positively affect mood states and performance. Significant improvements in mood and swim performance post-tapering suggest that such strategies can effectively mitigate the negative impacts of intense training (Aouani et al.). Moreover, a study on physical education and sports students explored the relationships between emotional intelligence, anxiety, and academic performance. High emotional intelligence was positively associated with better performance and lower anxiety levels, underscoring the importance of incorporating social and emotional learning programs in educational curricula to enhance student outcomes (Campos-Uscanga et al.). Exercise intervention positively affects emotional and cognitive health both psychologically and physiologically. A systematic review of fNIRS studies on exercise promoting brain health shows exercise interventions alter oxygenated hemoglobin levels in the prefrontal cortex and motor cortex, which are associated with improvements in higher cognitive functions (e.g., inhibitory control and working memory) (Shen et al., 2024). Another EEG study on exercise affecting creativity indicates that exercise in a natural environment is perceived subjectively differently from indoor exercise, participants report greater experiences of flow compared to indoor exercise, and the EEG measures objectively indicate enhanced cognitive activity in a creativity task after outdoor exercise (Kimura et al., 2023). Future research could focus on complex phenomena in athletes, such as choking under pressure, mindfulness has been used as an intervention for choking, using EEG neurofeedback training and other strategies might be useful (Hussey et al., 2020).

The body of research reviewed in these studies provides compelling evidence that exercise interventions can significantly enhance emotional health and cognitive functioning. However, the findings also point to the need for tailored strategies that consider individual differences, such as gender and personality traits, to maximize the benefits of physical activity (Herbert, 2022; Zhang et al., 2023). As we continue to explore these intricate relationships, it is crucial to develop and implement evidence-based interventions that promote both psychological and physiological well-being through exercise.

By synthesizing these findings, we can better understand the multifaceted benefits of exercise and pave the way for innovative approaches to enhance emotional and cognitive health in various populations.

## References

[B1] Herbert C. (2022). Enhancing mental health, well-being and active lifestyles of university students by means of physical activity and exercise research programs. Front. Public Health 10:849093. 10.3389/fpubh.2022.849093 35548074 PMC9082407

[B2] Hussey J. Weinberg R. Assar A. (2020). Mindfulness in sport: an intervention for a choking-susceptible athlete. Case Stud. Sport Exerc. Psychol. 4, 1–10. 10.1123/cssep.2019-002537711325

[B3] Kandola A. Stubbs B. (2020). “Exercise and anxiety,” in Physical Exercise for Human Health: Vol. 1228: Advances in Experimental Medicine and Biology, ed. J. J. Xiao (Cham: Springer Singapore), 345–352. 10.1007/978-981-15-1792-1_23 32342469

[B4] Kimura T. Mizumoto T. Torii Y. Ohno M. Higashino T. Yagi Y. (2023). Comparison of the effects of indoor and outdoor exercise on creativity: an analysis of EEG alpha power. Front. Psychol. 14:1161533. 10.3389/fpsyg.2023.1161533 37546462 PMC10400450

[B5] Mahindru A. Patil P. Agrawal V. (2023). Role of physical activity on mental health and well-being: a review. Cureus 15:e33475. 10.7759/cureus.33475 36756008 PMC9902068

[B6] Shen Q. Q. Hou J. M. Xia T. Zhang J. Y. Wang D. L. Yang Y.. (2024). Exercise promotes brain health: a systematic review of fNIRS studies. Front. Psychol. 15:1327822. 10.3389/fpsyg.2024.1327822 38659667 PMC11042249

[B7] Zhang Y. Li G. Liu C. Guan J. Zhang Y. Shi Z. (2023). Comparing the efficacy of different types of exercise for the treatment and prevention of depression in youths: a systematic review and network meta-analysis. Front. Psychiatry 14:1199510. 10.3389/fpsyt.2023.1199510 37333923 PMC10272399

